# Supplementary material for: A Python-Based Pipeline for Preprocessing LC–MS Data for Untargeted Metabolomics Workflows
Source: Metabolites. 2020 Oct 16;10(10):416. doi: 10.3390/metabo10100416 (PMC7602939; doi:10.3390/metabo10100416)
Supplement: Supplementary file 1 [file metabolites-10-00416-s001.pdf]

Supporting Information for

A Python-based pipeline for preprocessing LC-MS data for untargeted metabolomics workflows

Gabriel Riquelme,[1,2] Nicolás Zabalegui,[1,2] Pablo Marchi,[3] Christina M. Jones[4] and María Eugenia Monge[1*]

[1]Centro de Investigaciones en Bionanociencias (CIBION), Consejo Nacional de Investigaciones Científicas y Técnicas (CONICET), Godoy Cruz 2390, C1425FQD, Ciudad de Buenos Aires, Argentina.

[2]Departamento de Química Inorgánica Analítica y Química Física, Facultad de Ciencias Exactas y Naturales, Universidad de Buenos Aires, Ciudad Universitaria, C1428EGA, Buenos Aires, Argentina

[3]Facultad de Ingeniería, Universidad de Buenos Aires, Paseo Colón 850, C1063ACV, Ciudad de Buenos Aires, Argentina

[4]National Institute of Standards and Technology, Gaithersburg, MD, 20899-8392, USA.

*Corresponding author.

E-mail: maria.monge@cibion.conicet.gov.ar

Ph: +54 11 4899 5500 (ext. 5614)

Table of Contents

Section	Page
**Figure S1**: Extracted ion chromatograms for Phe-Phe, alogliptin, and Leu-Enk in the SSS	3
**Figure S2:** System suitability procedure for QC samples	4
**Figure S3:** PCA score plots for pooled QC samples	5
**Figure S4:** UML diagram for raw data preprocessing tools	6
**Figure S5:** UML diagram for data curation	7
**Supporting text 1:** Analytical batch correction	8
**Supporting text 2:** Feature Correspondence	9

Figure S1. Extracted ion chromatograms for Phe-Phe, alogliptin, and Leu-Enk in the SSS. Chromatographic peaks corresponding to the ten consecutive injections and used to establish the acceptance criteria are shown in blue. Those corresponding to the SSS analyzed before and after the study samples are shown in orange and green, respectively.

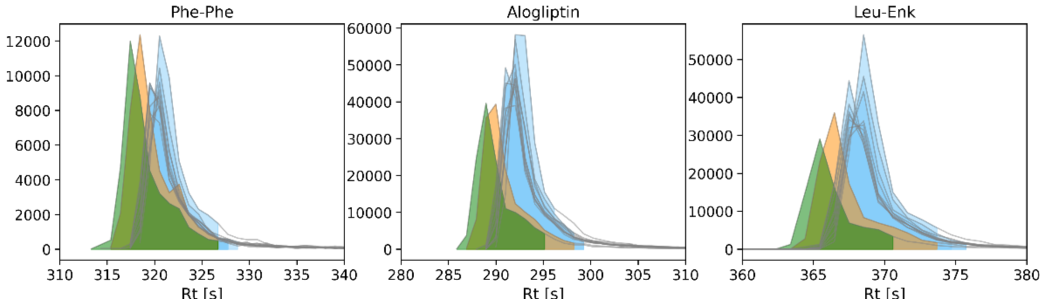

Figure S2. System suitability procedure: reproducibility of *m/z*, Rt and area values for pooled QC samples spiked with isotopically labeled leucine used as the internal standard, and with five authentic chemical standards used to prepare the system suitability QC sample (SSS). Values measured for process replicates were used to compute dispersion metrics for each analyte. (a) Dispersion for *m/z*, Rt and area for the internal standard and four chemical standards and (b) for LPC (18:0) that exhibited a much larger area than the other five compounds.

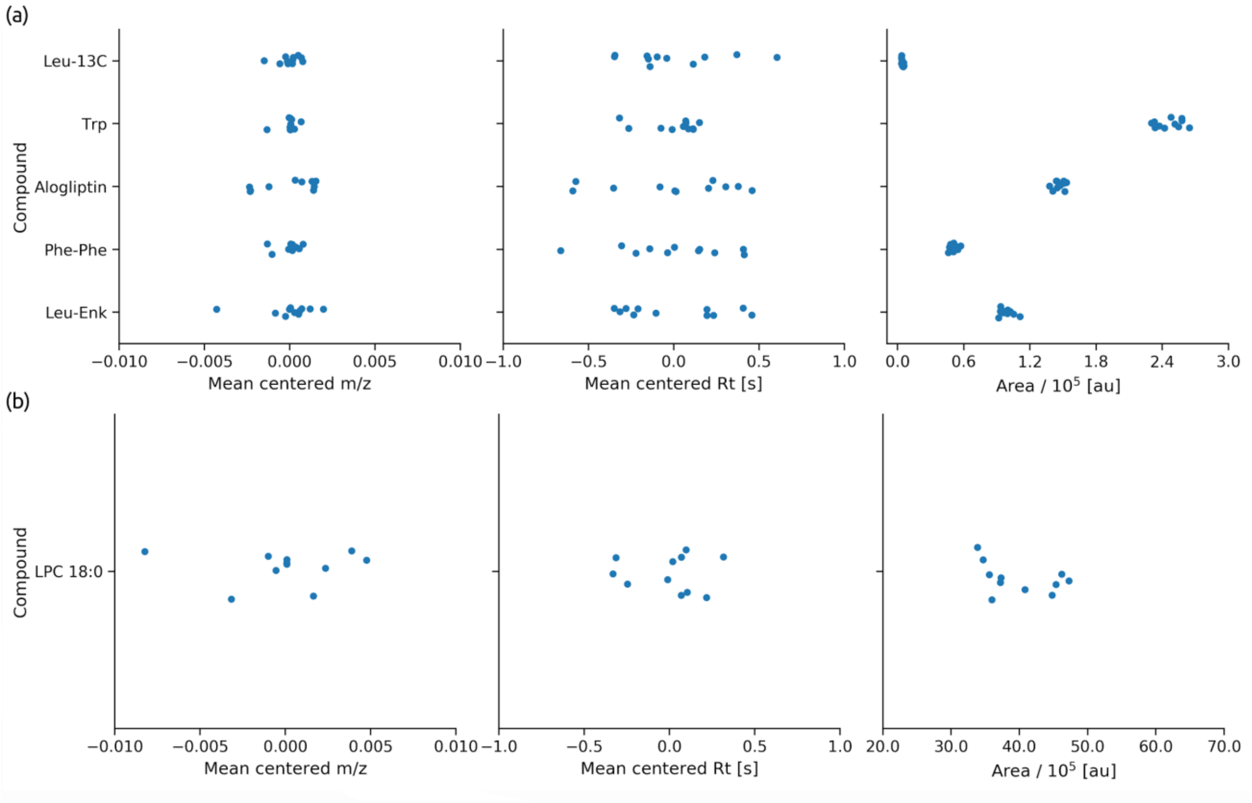

Figure S3. PCA score plots for pooled QC samples from models built: (a) before data curation and (b) after data curation. Data were normalized and autoscaled before PCA analysis.

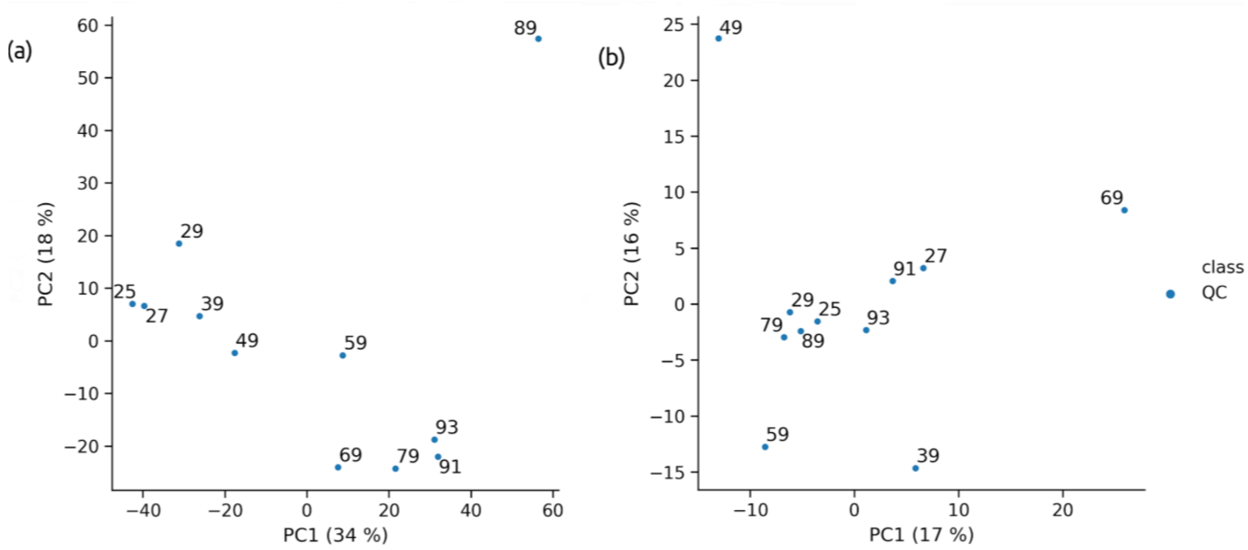

Figure S4. Unified Modelling Language (UML) diagram showing the organization and interaction between different objects used for reading and processing raw MS data in the package. Users can also check the API Reference available at Github [1].

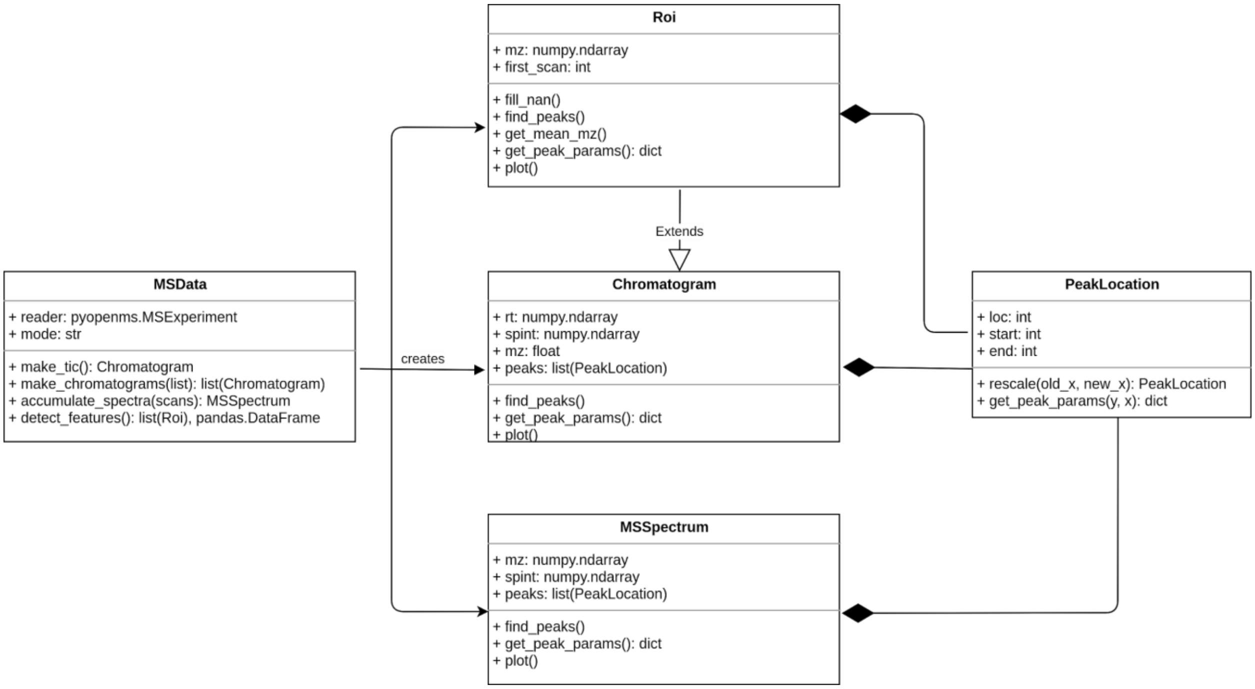

Figure S5. UML diagram showing interaction between different objects used for processing metabolomics data.

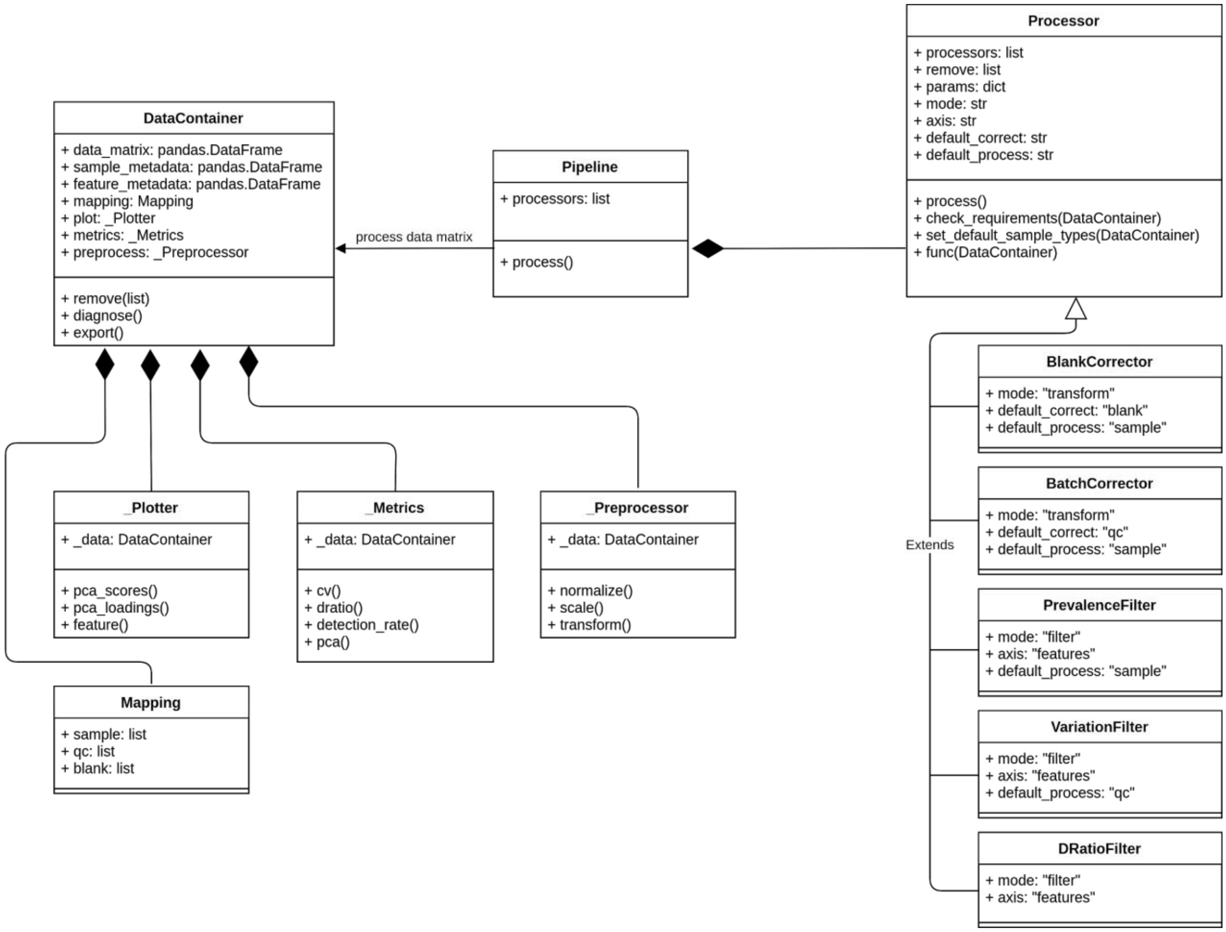

Supporting Text 1. Analytical batch correction

To correct the time dependent variation that can introduce bias in data analysis, we use a LOESS-based batch correction, as described by Dunn and collaborators in [2,3]. The aim of this supporting text is to discuss some considerations to implement this correction. We denote the data matrix M as a N × K matrix where N is the number of samples and K is the number of features. Each element of the matrix is denoted as $m_{i,j}$. The correction is based in using QC samples to estimate a time dependant systematic error function f_i for each i-th feature:

$$m_{i,j,QC} = \underline{m}_{i,QC} + f_i(t_{j,QC}) + \epsilon$$

Where $m_{i,j,QC}$ is the intensity of a QC in the data matrix associated to the i-th feature and j-th sample, $\underline{m}_{i,QC}$is the expected response in the QC for the i-th feature and ϵ is a noise term, that is assumed to be normal, independent and identically distributed (i.i.d.) for each sample.

The procedure to estimate f_i consists of several steps. The first step is to subtract $\underline{m}_{i,QC}$ to every QC sample. Then, we use LOESS to obtain a smoothed curve for f_i. Finally, we compute the values for each study sample using interpolation. One possibility to estimate $\underline{m}_{i,QC}$ would be to use the mean of QC values for feature i. However, if there is signal drift associated with carryover or sensitivity loss, the mean would be overestimating or underestimating $\underline{m}_{i,QC}$, respectively. Therefore, we have included the option to calculate $\underline{m}_{i,QC}$ using the first *n* QC samples of the batch or sample list to obtain a more accurate estimation of $\underline{m}_{i,QC}$.

Two verifications should be made in the data matrix before applying the LOESS-batch correction with the pipeline. First, the QC template used for sample analysis should include at least 4 points to perform the regression, but we suggest using at least five points to allow estimating the LOESS fraction parameter using leave-one-out cross validation (LOOCV). The second necessary consideration is to ensure that every study sample is "surrounded" by a QC sample within the sample list to allow interpolation of f_i. Features that do not meet these two conditions are removed. To avoid the removal of useful features we suggest using a QC template in each untargeted metabolomics study with several QC samples at the beginning and at the end of each analytical batch (three are recommended). After correcting each analytical batch, a global mean between all batches is computed and each batch is transformed to share the same mean, subtracting the batch mean and adding the global mean.

Supporting Text 2. Feature Correspondence

The feature correspondence algorithm implemented in the package and used in the Application #1 is described based on its requirements and the implementation. In addition, the algorithm is tested in cases with simulated data. We plan to continue the development of this method to allow for feature correspondence of complex untargeted metabolomics data.

Requirements

Several considerations should be taken into account when performing feature correspondence in LC-MS data [4]. For a correspondence algorithm to be effective, we need to consider the different causes of dispersion in *m/z* and Rt, as well as the characteristics of the data sets in metabolomics. For modelling LC-MS data, we are going to assume that:

1. *m/z* dispersion for a given ionic species is mostly associated with the precision of the mass spectrometer.
2. Rt dispersion depends not only on the analytical performance of the chromatographic system, but also is affected among others by concentration levels, ionization efficiency and matrix effects that may be different for each metabolite in the sample.
3. *m/z* and Rt dispersion are independent.
4. Features are not necessarily detected in all samples.

Typical limitations encountered in other methods for feature correspondence include the use of a reference sample, and the use of alignment-based methods. The former causes asymmetric solutions depending on the sample chosen as reference, whereas the latter usually works with binned data in the *m/z* dimension and fails to capture the retention time variation of each species in the sample.

Taking into consideration these points, we have implemented a clustering-based approach using the feature descriptors obtained during feature detection. This approach has also been used in other works [5,6], but with different strategies from ours. The descriptors include *m/z*, Rt, the sample in which the feature was detected, and the sample class. The aim of the approach is to

build feature clusters with each feature being generated from the same ionic species. Minimum requirements for a **valid cluster** include similar *m/z* and Rt values, and that only one feature from a given sample should be present in each cluster. These conditions may not be accomplished in the following cases:

1. if artifacts are detected as features during the feature detection process;
2. if features from ionic species are present in a low number of samples and have similar Rt and *m/z* values;
3. if more than one ionic species is present in the cluster, for example when different species have similar *m/z* and Rt values.

Cases #1 and #2 address **repeated features** and they need to be removed from the data. For case #3, the cluster should be split according to the number of species that are present there. The minimum number of features n_{min} that defines a cluster needs to be considered as well, since a cluster with less than n_{min} samples would be discarded. Overall, clusters are created from the features detected in each sample, and each cluster is analyzed for splitting if more than one species is present, or for removing repeated features.

Initial feature clustering

The DBSCAN algorithm [7] is used for the initial data clustering step because it is a clustering algorithm that can detect clusters with arbitrary shapes as well as noisy points in the data. DBSCAN builds clusters using an ϵ parameter to connect points: two points are connected if their distance is lower than ϵ. Each point in the data is classified in one of the following three categories according to the number of points that are connected to it:

- Core, if it has at least n_{min}neighbours, including itself
- Reachable, if it is connected to a core point
- Noise, otherwise.

A cluster has at least one core point, and points are connected using the *distance* function. To cluster LC-MS features and capture most of the data behaviour, the parameters ϵ, *distance*, and

n_{min} need to be defined. The ϵ parameter is set based on the expected dispersion in *m/z* and Rt. The dispersion ϵ_{mz} is mostly related to the instrument precision. For Rt dispersion, a possibility is to fix a maximum time window for all peaks or build an initial clustering of the data based on the closeness of the points and then analyze each cluster separately. With this consideration in mind, an ϵ_{rt} of 5 s was selected to account for features with high Rt dispersion variation for UHPLC measurements. Before feature clustering, the Rt of each feature must be scaled to be jointly analyzed with *m/z* as follows:

$$Rt_{scaled} = Rt\frac{\epsilon_{mz}}{\epsilon_{Rt}}$$

The Chebyshev distance is used because the dispersions in Rt and *m/z* dimensions are independent. To set the n_{min} parameter, the fraction of samples in a class f_{class} is defined as the ratio between samples in a class and the total number of samples as follows:

$$f_{class} = \frac{n_{class}}{n_{samples}}$$

Using this value, the parameter *min_f* varies between 0 and 1 and is used to estimate n_{min} as the product between *min_f* and the number of samples in the class with lowest f_{class}. After this step, clusters of features with similar Rt and *m/z* are obtained, and features that do not have neighbors are classified as noise and removed from the data. Finally, each cluster needs to be assessed.

Cluster assessment

Once clusters are created they must be validated. The first step is to test how many species are included in a cluster. To do this, we count the number of k-repetitions, $n_{k,rep}$ present in a cluster, e.i., the number of times a sample contributes with k features. The number of species, n_s in a cluster is estimated as the largest value of k such that $n_{k,rep}$ is larger than n_{min}. Using the number of species, each cluster is fitted into a Gaussian Mixture Model (GMM) using n_s components. Once again, because the *m/z* and Rt dispersions are independent, the covariance matrices used to build the GMM are restricted to diagonal matrices. Using the GMM on each cluster, subclusters can be created assigning each feature to a component of the GMM, and

removing repeated features. To assign each feature to a subcluster, features are grouped using the sample in which they were detected. If the number of features is larger than the number of species, the score for each feature (computed as the log-likelihood in the model) is evaluated and features with the lowest score are removed until the number of features is equal to n_s. Subsequently, n_s features need to be assigned to n_s subclusters. This problem can be solved using the Hungarian algorithm with a cost matrix where each row is a feature and each column is the posterior probability of a component in the GMM. If there are less than n_s features, the cost matrix is completed with rows of zeros. After this step, clusters of valid features are obtained. Finally, the GMM is used to recover missing features, that is, features from samples that contributed zero features to the cluster. During the initial data clustering step, a feature can be flagged as noise if their *m/z* and Rt values are outside the tolerance value initially set. To recover missing features, features flagged as noise by DBSCAN (or repeated features that were removed from other clusters) and that come from missing samples are fitted to the GMM. To include them in the cluster, the *min_likelihood* parameter can be used, and a feature is recovered only if its likelihood is larger than this parameter.

Preliminary results

To test how this method can match features, we analyzed two simulated overlapping species using a bivariate normal distribution for each one and evaluated the match accuracy in cases where the *m/z* and Rt difference between each species was close to ϵ_{mz} and ϵ_{rt}, respectively. Four different conditions were tested using different parameters for each distribution. For each condition, 100 samples were generated from each distribution and one of the distributions was fixed using a mean vector of (100.0100, 120) (values are in *m/z* and Rt units), and a standard deviation vector of (0.0025, 1) (both gaussian distributions had diagonal covariance matrices). The parameters for the other distribution were varied in each condition (see Table S1). The following parameters were used for feature correspondence: $\epsilon_{mz} = 0.005$, $\epsilon_{rt} = 5$, *min_fraction = 0.2, min_likelihood = 0* . Figure S5 illustrates the dispersion for each data set and how each feature was matched. The results show features with high accuracy can be matched even in cases where there is a high degree of overlap between the distributions used.

This algorithm was successfully used to match the features in Application 1 with 100 % accuracy, which was manually verified since these features addressed a set of known chemical standards. Based on the tests performed in simulated data, we believe that this method has the potential to be used on untargeted metabolomics data sets that typically present peak overlap and features present larger retention time variation. However, more testing needs to be done. Indeed, the inclusion of other feature descriptors in the analysis, such as charge, isotopic distribution and peak area, would allow the correct assignment of features in those cases in which *m/z* and Rt information is not enough to confidently assign features to a cluster.

Table S1. Parameters used for the normal distribution of species 1. Both μ and σ are a vector of *m/z* and Rt. The third column shows the accuracy for the feature correspondence in each condition.

Set	μ	σ	Accuracy (%)
0	100.0200, 125	0.0025, 3	100
1	100.0150, 125	0.0025, 3	100
2	100.0200, 123	0.0025, 3	99
3	100.0150, 123	0.0025, 3	94

Figure S6. *m/z* and Rt dispersion for the simulated features in each set. The color for each point shows the distribution from where they were sampled, while the marker shows the cluster to which each feature was assigned. Even when there is a high overlap, as in sets #2 and #3, the classification accuracy is close to 1.

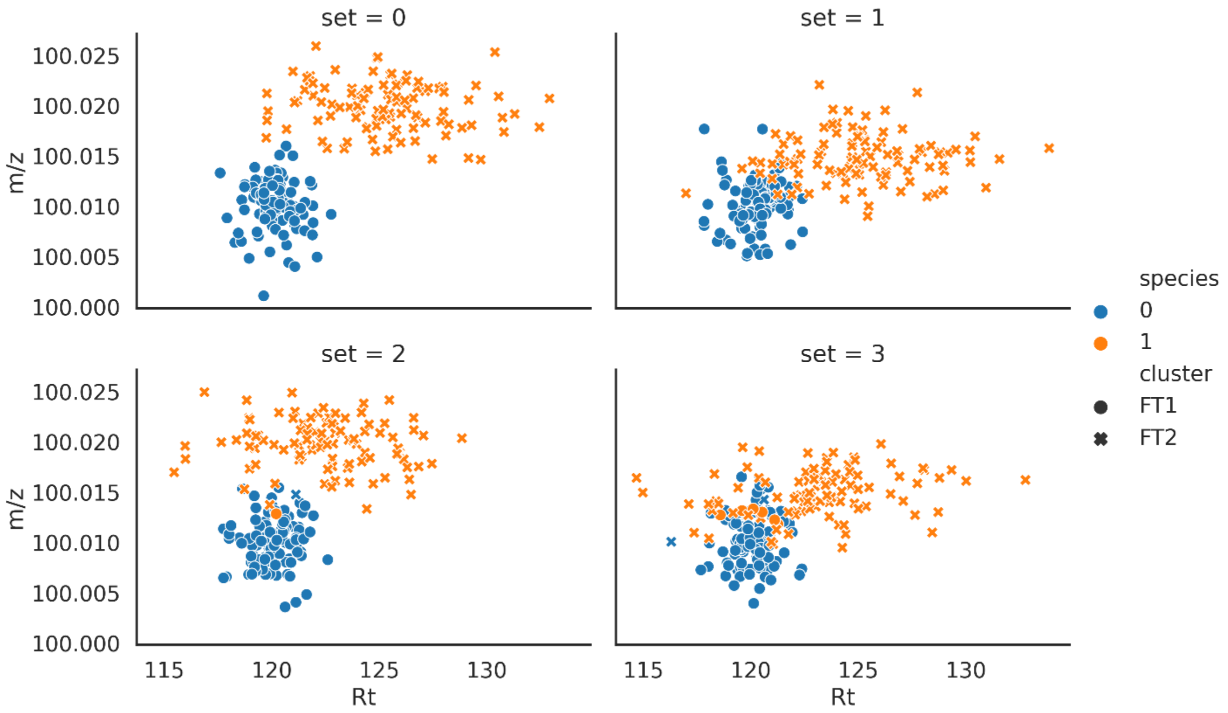

References for Supporting Information

1. TidyMS Repository Available online: https://github.com/griquelme/tidyms.
2. Dunn, W.B.; Broadhurst, D.; Begley, P.; Zelena, E.; Francis-McIntyre, S.; Anderson, N.; Brown, M.; Knowles, J.D.; Halsall, A.; Haselden, J.N.; et al. Procedures for large-scale metabolic profiling of serum and plasma using gas chromatography and liquid chromatography coupled to mass spectrometry. *Nat Protoc* **2011**, *6*, 1060–1083, doi:10.1038/nprot.2011.335.
3. Broadhurst, D.; Goodacre, R.; Reinke, S.N.; Kuligowski, J.; Wilson, I.D.; Lewis, M.R.; Dunn, W.B. Guidelines and considerations for the use of system suitability and quality control samples in mass spectrometry assays applied in untargeted clinical metabolomic studies. *Metabolomics* **2018**, *14*, doi:10.1007/s11306-018-1367-3.
4. Smith, R.; Ventura, D.; Prince, J.T. LC-MS alignment in theory and practice: a comprehensive algorithmic review. *Brief Bioinform* **2015**, *16*, 104–117, doi:10.1093/bib/bbt080.
5. Smith, C.A.; Want, E.J.; O'Maille, G.; Abagyan, R.; Siuzdak, G. XCMS: processing mass spectrometry data for metabolite profiling using nonlinear peak alignment, matching, and identification. *Anal. Chem.* **2006**, *78*, 779–787, doi:10.1021/ac051437y.
6. Alkhalifah, Y.; Phillips, I.; Soltoggio, A.; Darnley, K.; Nailon, W.H.; McLaren, D.; Eddleston, M.; Thomas, C.L.P.; Salman, D. VOCCluster: Untargeted Metabolomics Feature Clustering Approach for Clinical Breath Gas Chromatography/Mass Spectrometry Data. *Anal. Chem.* **2020**, *92*, 2937–2945, doi:10.1021/acs.analchem.9b03084.
7. Ester, M.; Kriegel, H.-P.; Sander, J.; Xu, X. A density-based algorithm for discovering clusters in large spatial databases with noise. In Proceedings of the Proceedings of the Second International Conference on Knowledge Discovery and Data Mining; AAAI Press: Portland, Oregon, 1996; pp. 226–231.
